# Supplementary material for: In Silico Analysis of Post-COVID-19 Condition (PCC) Associated SNP rs9367106 Predicts the Molecular Basis of Abnormalities in the Lungs and Brain Functions
Source: Int J Mol Sci. 2025 Jul 11;26(14):6680. doi: 10.3390/ijms26146680 (PMC12294317; doi:10.3390/ijms26146680)
Supplement: Supplementary file 1 [file ijms-26-06680-s001.zip › ijms-3701431-supplementary.pdf]

Supplementary Information

In Silico Analysis of Post-Covid-19 Condition (PCC) Associated SNP rs9367106 Predicts the Molecular Basis of Abnormalities in the Lungs and Brain Functions

Amit K Maiti[1]*

[1] Department of Genetics; Mydnavar,28475 Greenfield Rd, MI USA, amit.maiti@mydnavar.com; akmit123@yahoo.com

* Correspondence: amit.maiti@mydnavar.com; Tel.: +1 248 379 3129)

Supplementary Figure S1: Compartment analysis of rs9367106 -*FOXP4* region in Chr6 at 500kb resolution in Mi-C dataset (Krietenstein et al, 2020). Both the SNP and *FOXP4* belong in the same compartment designated by two arrows.

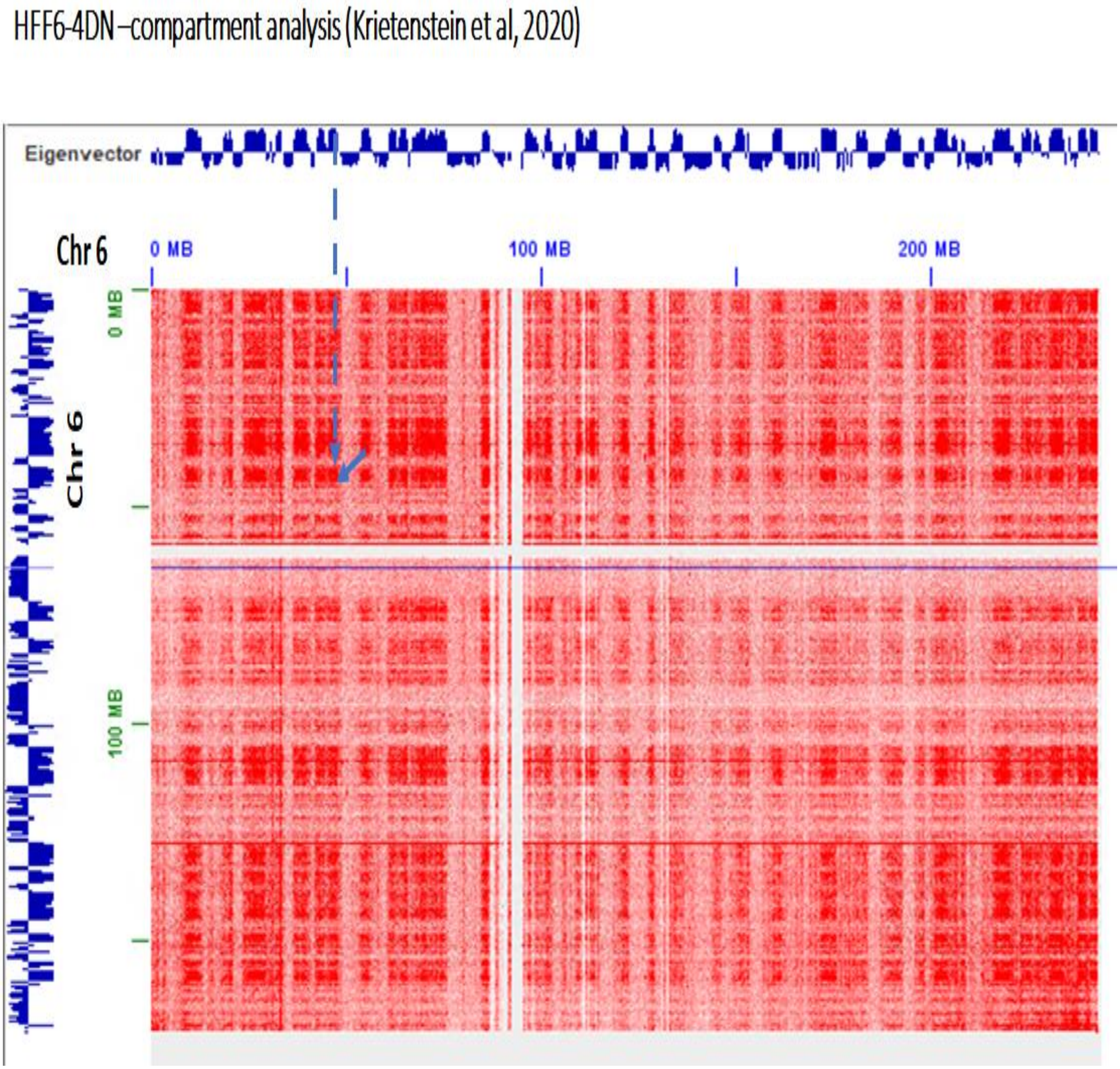

Supplementary Figure S2: Chia-PET physical interaction of rs9367106 with *FOXP4* and in other distant genes.

Chia-pet at rs9367106-FOXP4 with POL2RA Data (Ruan lab)

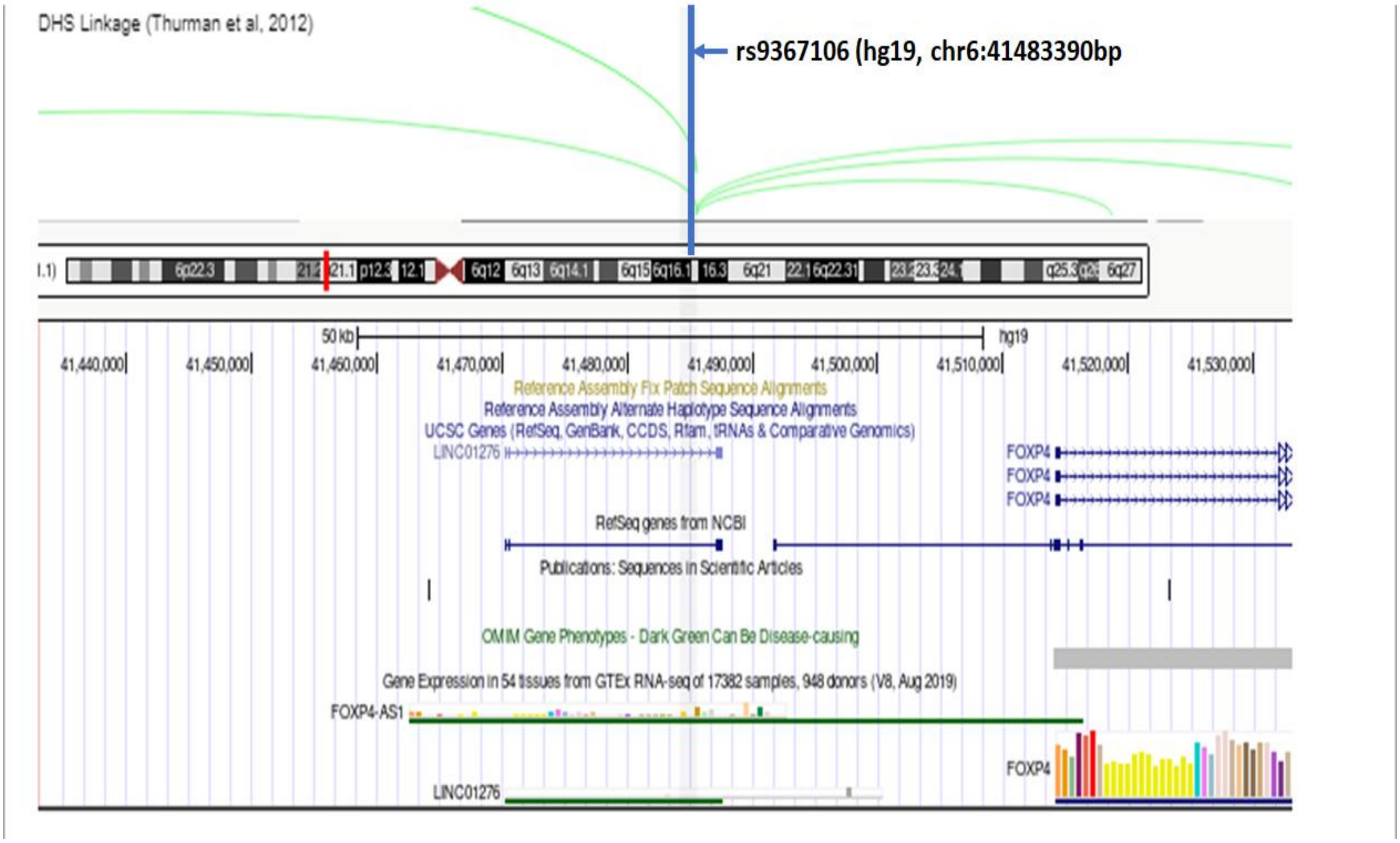

Supplementary Figure S3: Chia-PET physical interaction of rs9367106/*LINC01276* with *MED20* gene promoter

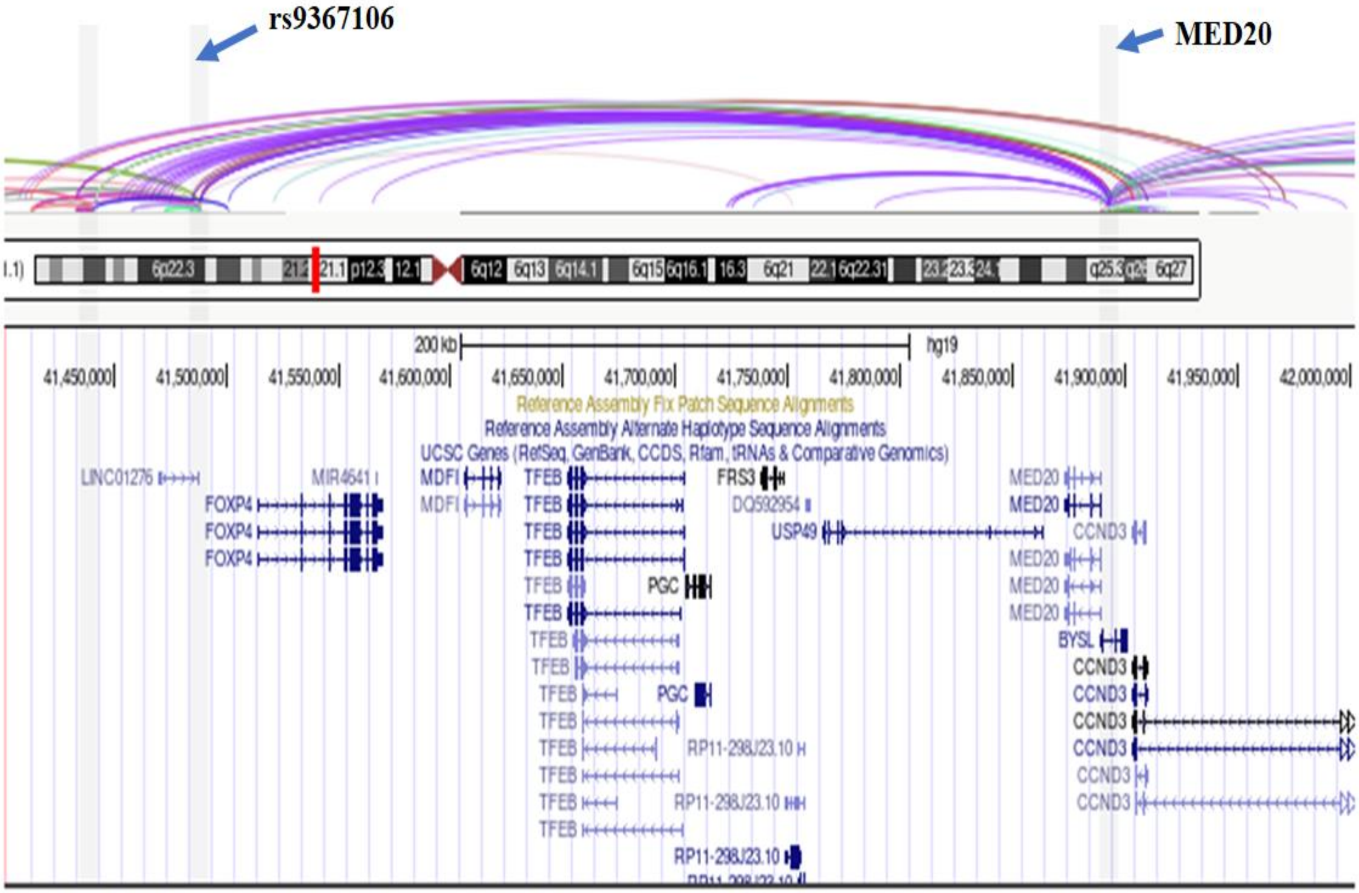

Supplementary Figure S4: Expression of *LINC01276* in various human tissue. . RNA-seq experiments indicate it expresses maximally in Lung and testis. It also expresses in various brain tissues moderately.

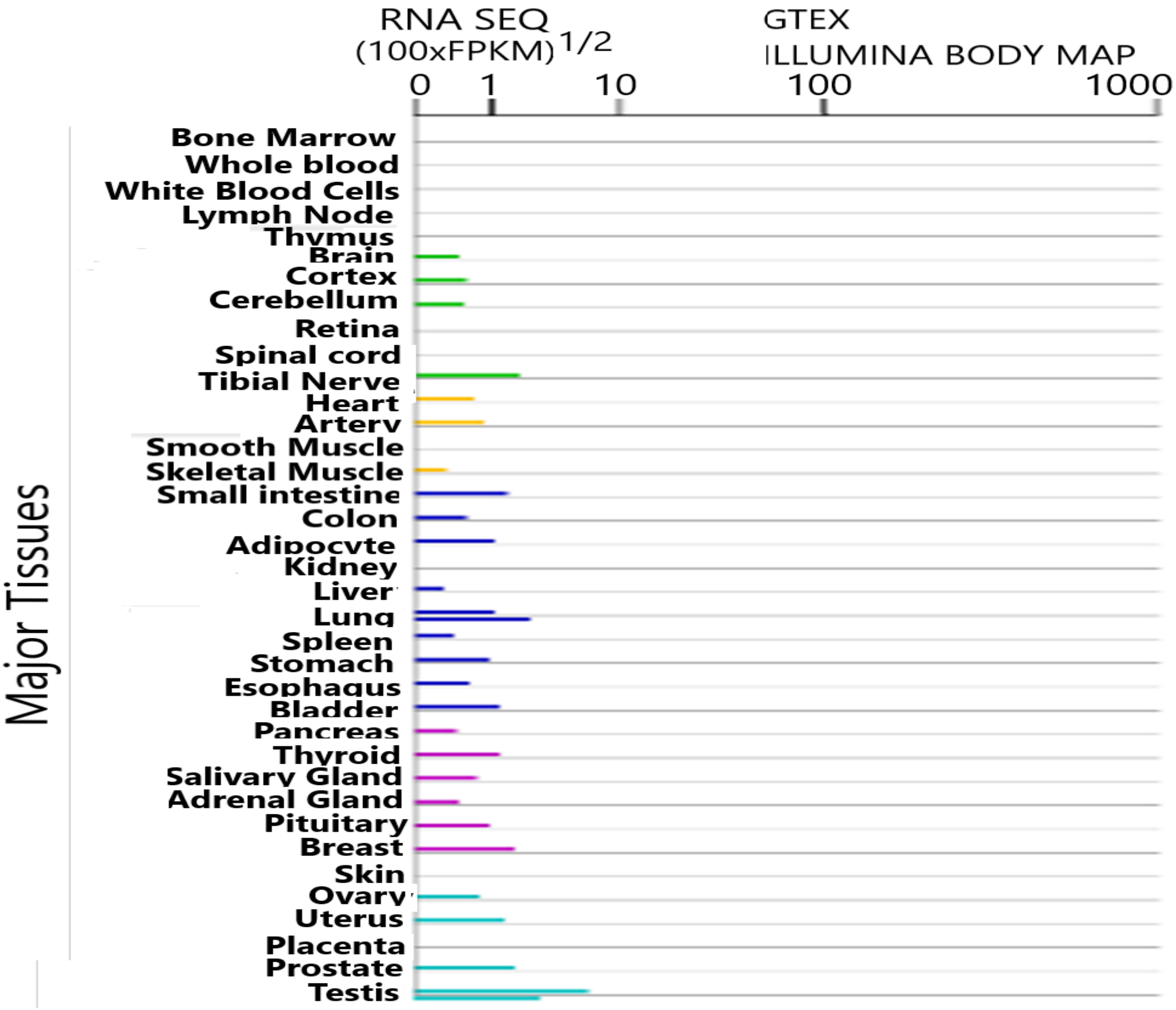

Supplementary Figure S5: Tissue specific expression of *MED20* in human. *MED20* expresses many tissues including lung and brain. It especially expresses in cortex, spinal nerves, tibial nerves and hypothalamus.

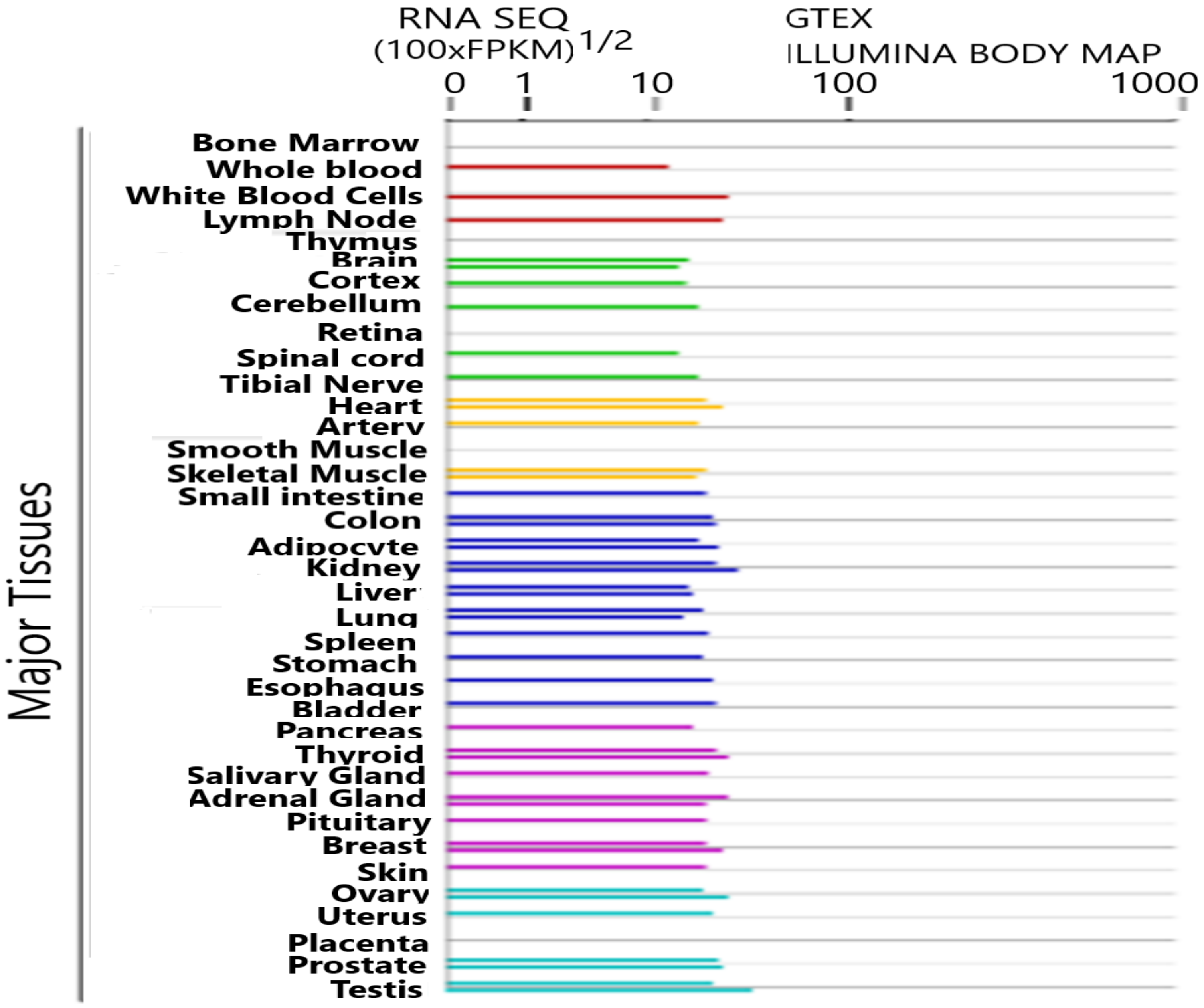

Supplementary Figure S6: Expression of *FOXP4-AS1* in human tissue. *FOXP4-AS1* is expressed mainly in heart, kidney, colon, liver thus unlikely to play major role in LongCOVID-19 syndrome.

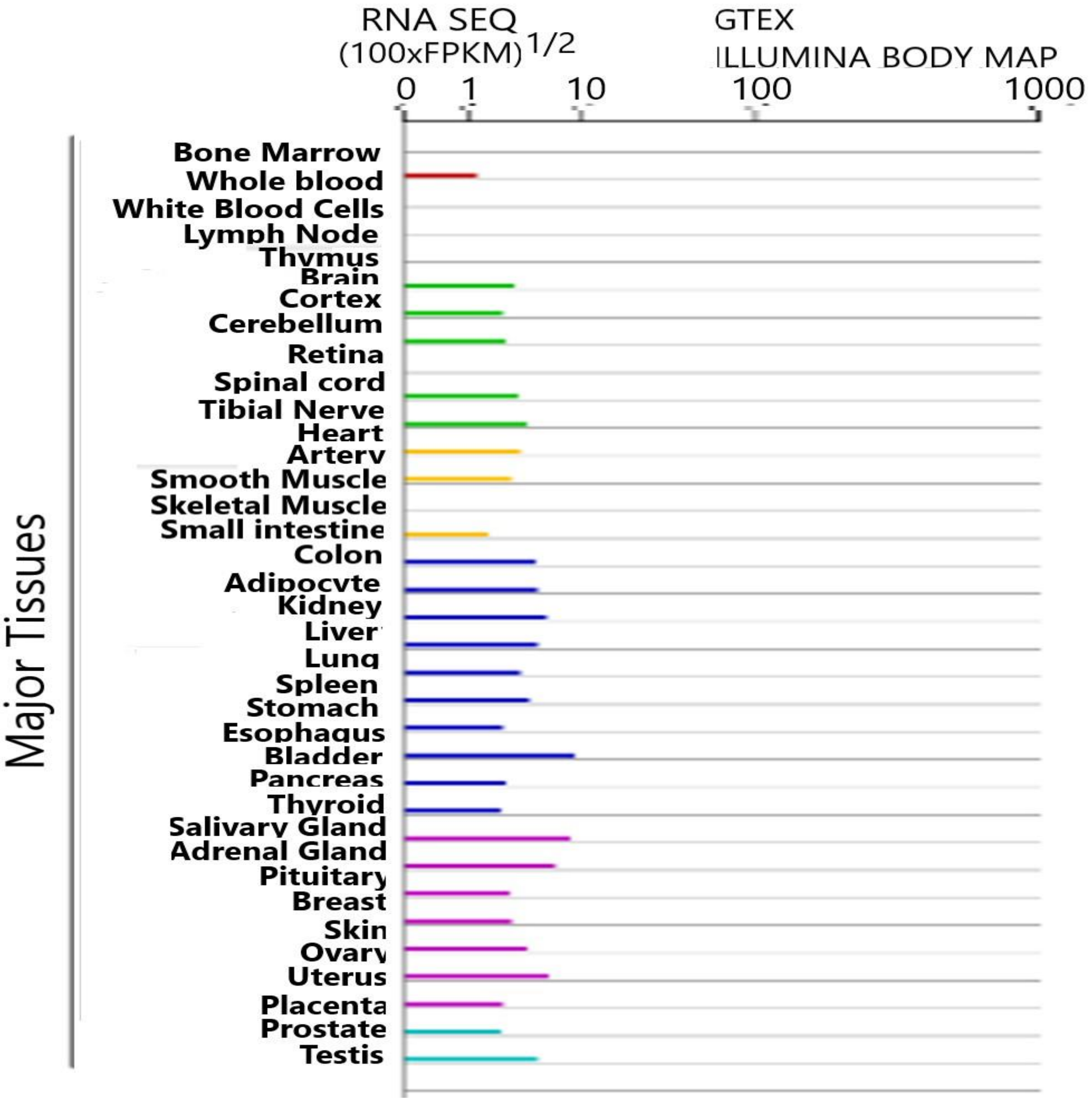

Supplementary Figure S7: Expression of *FOXP4* mRNA and protein in various human tissues

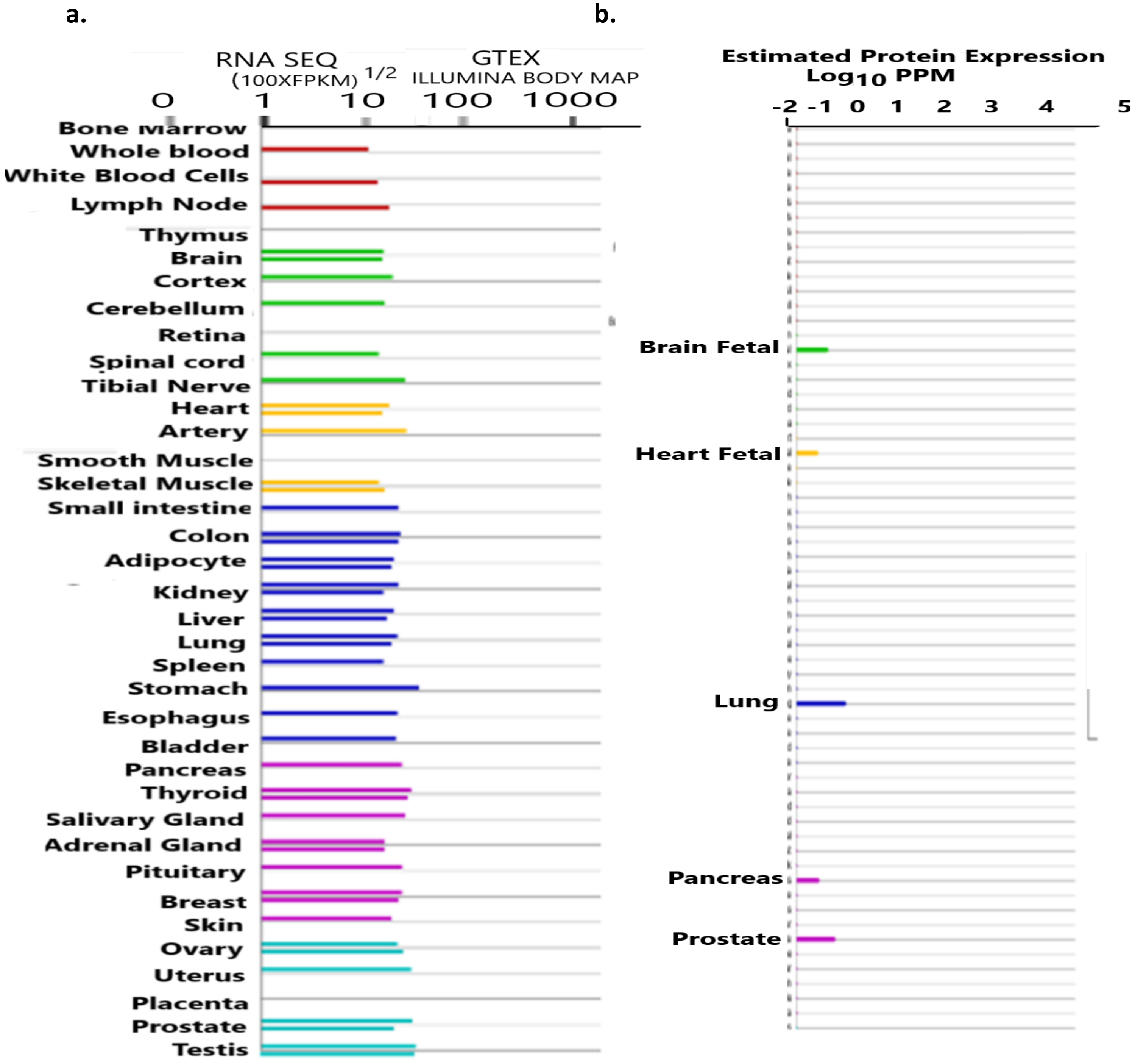

Supplementary Figure S8. Single cell atlas of expression of *FOXP4* in various lung cells, especially in alveolar cells (c0)

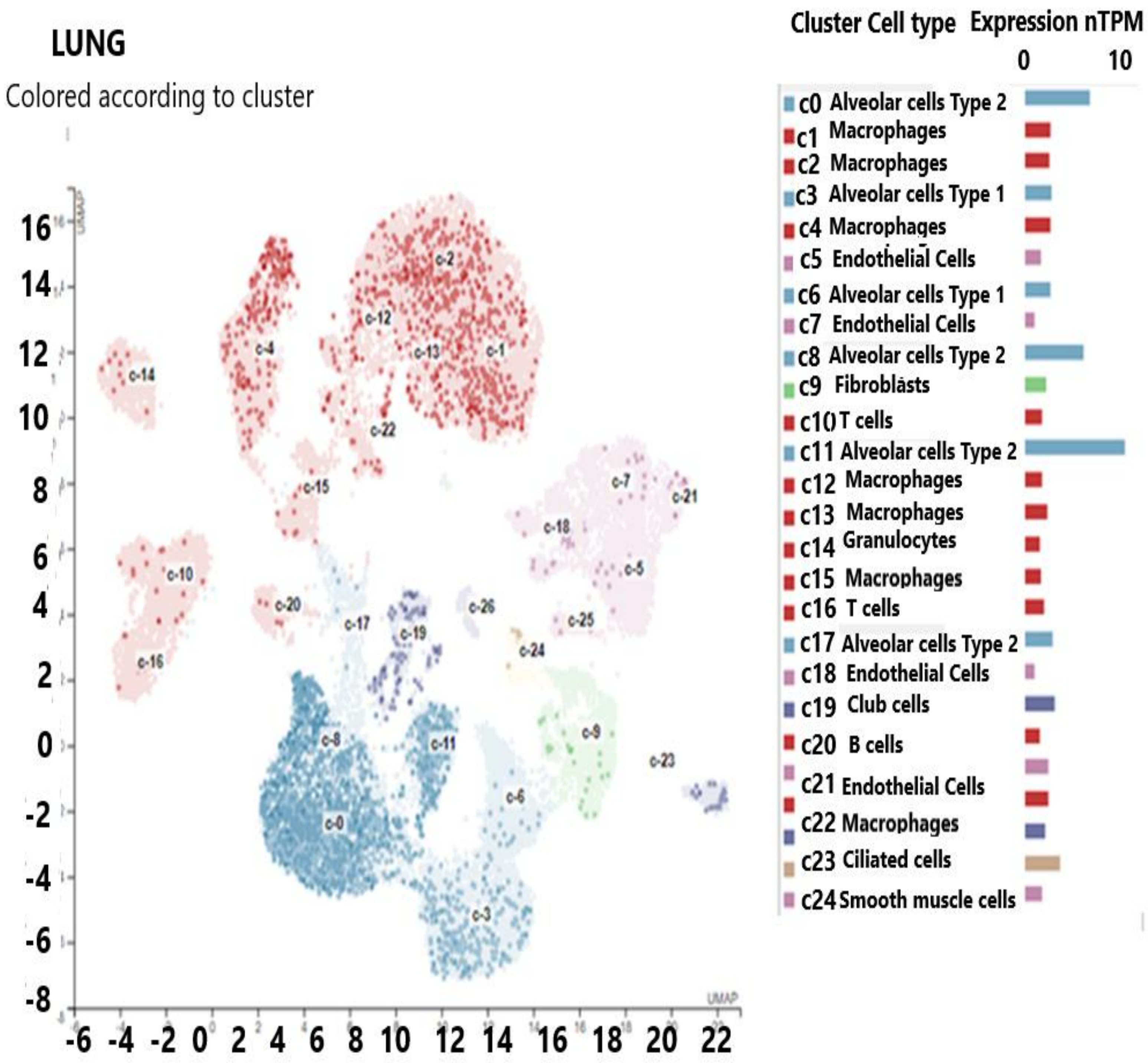

Supplementary Figure S9: Single cell atlas of the expression of *FOXP4* in various brain cells.

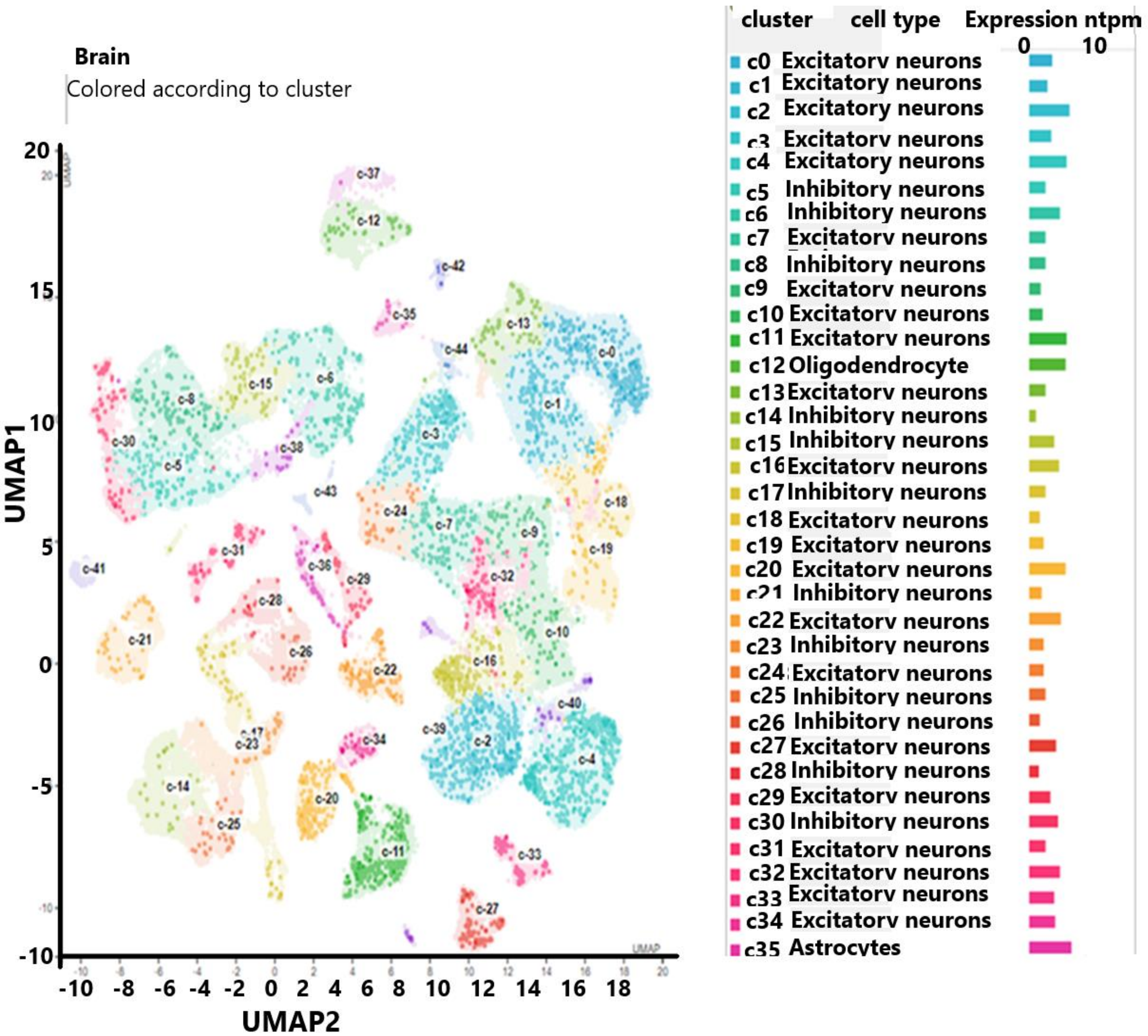

Supplementary Figure S10. Gene network analysis of FOXP4. FOXP4 interacts with FOXP1 and SOX2 axis to modulate functions in brain and lungs.

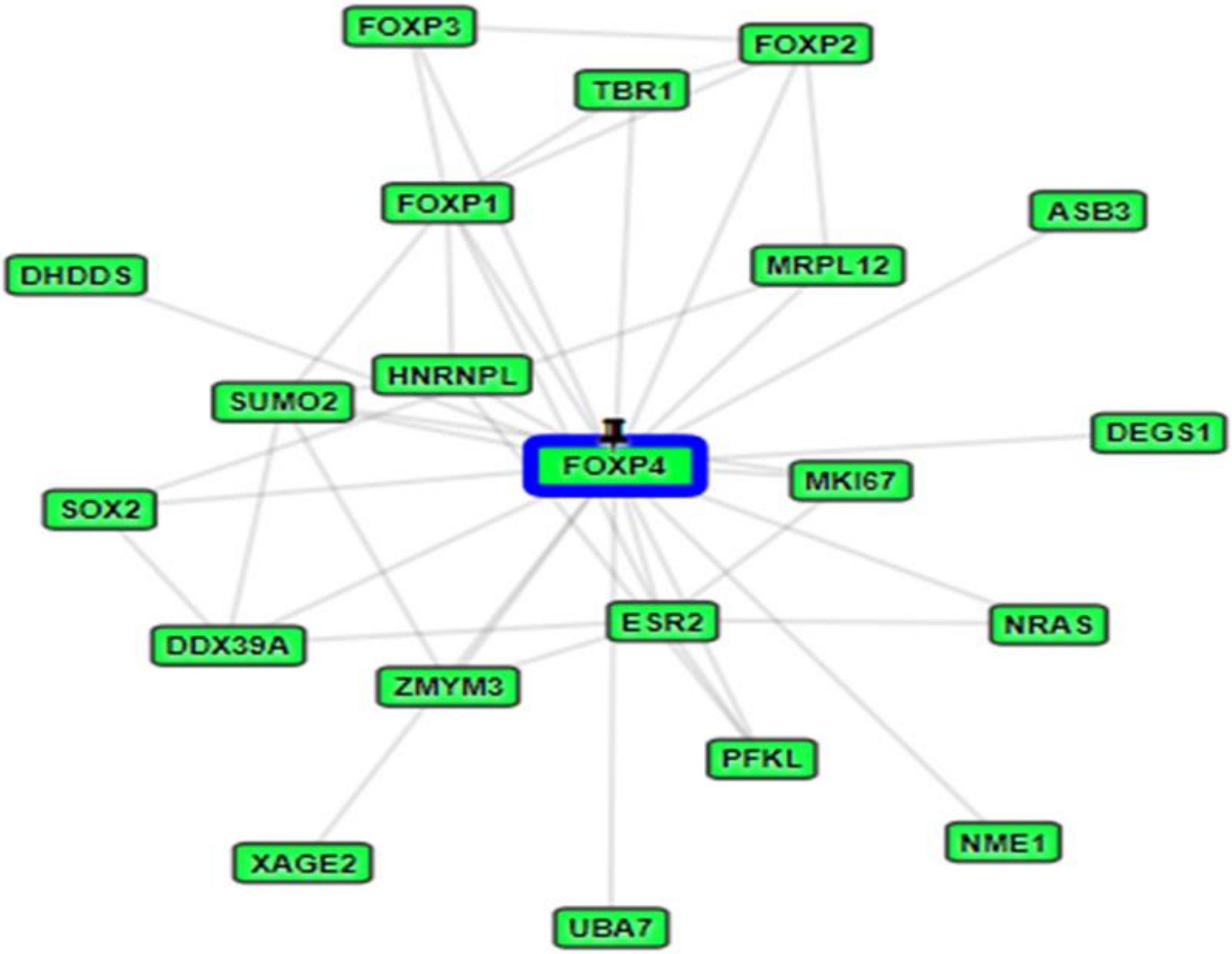

Supplementary Figure S11. Expression and interaction of rs9367106 co-expressed Geneset in various tissues and brain tissues

a

Index	Name	P-value	Adjusted p-value	Odds Ratio	Combined score
1	HT55 LARGE INTESTINE TenPx06	0.05690	0.8878	3.66	10.48
2	HCC1937 BREAST TenPx18	0.07730	0.8878	3.20	8.20
3	PC14 LUNG TenPx05	0.08491	0.8878	4.32	10.66
4	HCC95 LUNG TenPx11	0.1026	0.8878	3.86	8.78
5	MKN45 STOMACH TenPx17	0.1144	0.8878	3.61	7.82
6	LS180 LARGE INTESTINE TenPx27	0.1305	0.8878	2.52	5.12
7	NCIH1666 LUNG TenPx16	0.1329	0.8878	2.50	5.04
8	CL34 LARGE INTESTINE TenPx38	0.1401	0.8878	2.43	4.78
9	RMUGS OVARY TenPx31	0.1449	0.8878	3.11	6.00
10	RERFLCMS LUNG TenPx01	0.1625	0.8878	5.84	10.62

b

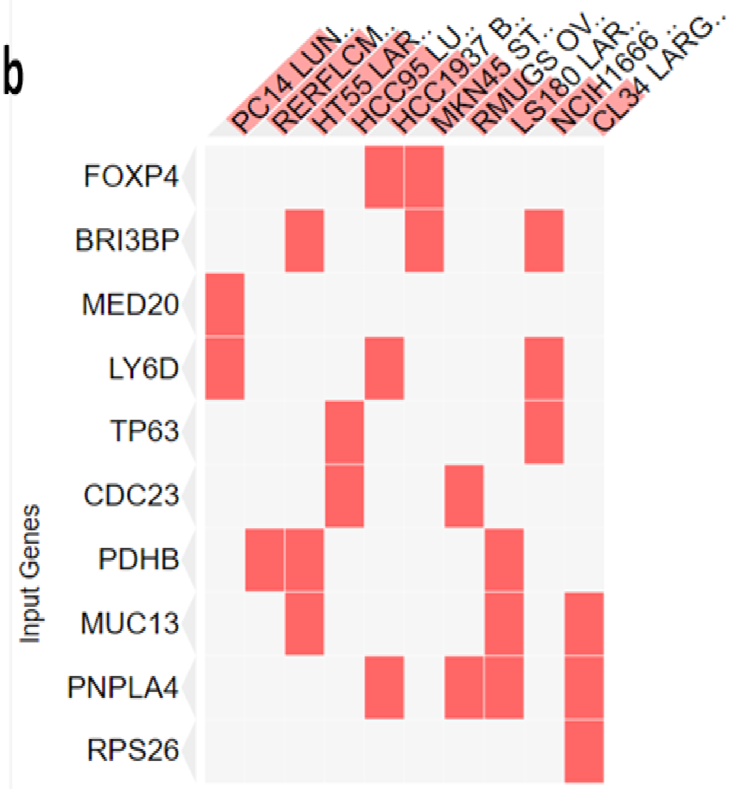

c

Index	Name	P-value	Adjusted p-value	Odds Ratio	Combined score
1	mantle zone of r9BM	0.01535	0.4242	6.21	25.93
2	medial part of r9B	0.01535	0.4242	6.21	25.93
3	superficial stratum of r10BM	0.09661	0.4242	4.00	9.35
4	intermediate stratum of r9BM	0.09661	0.4242	4.00	9.35
5	periventricular stratum of p1Lim	0.09661	0.4242	4.00	9.35
6	Arcuate hypothalamic nucleus	0.09661	0.4242	4.00	9.35
7	superficial stratum of r9BI	0.09661	0.4242	4.00	9.35
8	superficial stratum of r9BM	0.09661	0.4242	4.00	9.35
9	superficial stratum of the Fc	0.09661	0.4242	4.00	9.35
10	ventral part of THyB	0.09661	0.4242	4.00	9.35

Showing 1 to 10 of 258 entries | Export entries to table

Previous Next

Terms marked with an * have an overlap of less than 5

d

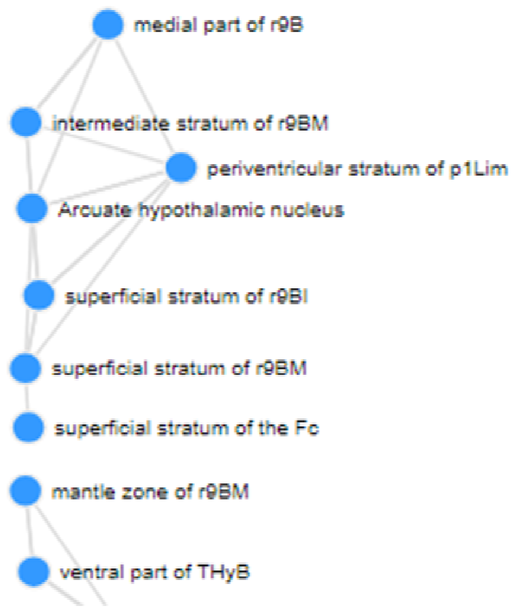

Supplementary Table S1: rs9367106 co-expressed genes in ARCH4 RNA seq database Genes

MRPL35P2
BRI3BP
RNA5SP510
SLC25A1P4
RNU6ATAC32P
RNA5SP512
FOXP4
TERB1
TRGV11
RPS26
MED20
PNPLA4
LY6D
RN7SL552P
RNU6-891P
ACOT7
PCBP2P3
RPS7P12
ANKRD11P1
LINC01276
PRR11-AS1
PDHB
FOXP4-AS1
GPALPP1
HMGB1P10
FAM9CP1
NPM1P6
CDC23
PDCL3P3
MUC13
SCML2P1
TRDC
GRAMD4P1
ZNF345
TP63
